# Applying Unconventional Secretion in *Ustilago maydis* for the Export of Functional Nanobodies

**DOI:** 10.3390/ijms18050937

**Published:** 2017-04-29

**Authors:** Marius Terfrüchte, Michèle Reindl, Silke Jankowski, Parveen Sarkari, Michael Feldbrügge, Kerstin Schipper

**Affiliations:** 1 Institute for Microbiology, Cluster for Excellence on Plant Sciences, Heinrich Heine University Düsseldorf, 40204 Düsseldorf, Germany; marius.terfruechte@hhu.de (M.T.); michele.reindl@hhu.de (M.R.); silke.jankowski@hhu.de (S.J.); feldbrue@hhu.de (M.F.); 2 Bioeconomy Science Center (BioSC), c/o Forschungszentrum Jülich, 52425 Jülich, Germany; 3 Department of Biotechnology (DBT), Muthgasse 18, 1190 Vienna, Austria; parveen.sarkari@boku.ac.at

**Keywords:** *Ustilago maydis*, unconventional secretion, nanobody, chitinase, botulinum toxin A

## Abstract

Exploiting secretory pathways for production of heterologous proteins is highly advantageous with respect to efficient downstream processing. In eukaryotic systems the vast majority of heterologous proteins for biotechnological application is exported via the canonical endoplasmic reticulum–Golgi pathway. In the endomembrane system target proteins are often glycosylated and may thus be modified with foreign glycan patterns. This can be destructive for their activity or cause immune reactions against therapeutic proteins. Hence, using unconventional secretion for protein expression is an attractive alternative. In the fungal model *Ustilago maydis*, chitinase Cts1 is secreted via an unconventional pathway connected to cell separation which can be used to co-export heterologous proteins. Here, we apply this mechanism for the production of nanobodies. First, we achieved expression and unconventional secretion of a functional nanobody directed against green fluorescent protein (Gfp). Second, we found that Cts1 binds to chitin and that this feature can be applied to generate a Gfp-trap. Thus, we demonstrated the dual use of Cts1 serving both as export vehicle and as purification tag. Finally, we established and optimized the production of a nanobody against botulinum toxin A and hence describe the first pharmaceutically relevant target exported by Cts1-mediated unconventional secretion.

## 1. Introduction

Heterologous proteins are preferentially produced in systems in which they are exported into the culture broth, as this simplifies downstream processing and minimizes the production costs substantially [1]. In eukaryotic expression systems secretion is usually achieved via the canonical endoplasmic reticulum (ER)–Golgi pathway. Characteristic signal peptides located at the amino terminus determine the cargo for this route at the beginning of translation and mediate its co-translational uptake into the ER. Folding and protein modifications like *N*-glycosylation take place during passage of the intermembrane system. Finally, proteins are released to the cell exterior by the fusion of secretory vesicles with the plasma membrane [2,3].

Various well-established expression systems exist, often providing high space-time yields [4,5]. These include for example bacterial systems like *Escherichia coli* or *Bacillus subtilis* in which mostly unmodified proteins are produced [6,7,8]. The yeasts *Saccharomyces cerevisiae* and *Pichia pastoris*, or filamentous fungi like *Aspergilli* are established workhorses mainly for generating eukaryotic proteins [9,10,11]. For production of expensive therapeutic proteins, human or CHO cell cultures are preferentially used, especially if post-translational modifications are required for protein function [5,12]. However, these cultures are more costly, slow growing, and it is elaborate and time-consuming to develop expression lines [4]. Eukaryotic cell-free systems are emerging as an alternative, but are also expensive and only support post-translational modifications in some cases [13]. The choice of an expression system strongly depends on the requirements of the target protein. If a protein can easily be expressed in one of the established systems, this is the preferred production host. However, many desired proteins are still very difficult to produce. Therefore, alternative production strategies are in high demand.

In distinct cases, sending heterologous proteins of interest via the conventional eukaryotic secretion pathway can cause severe problems because they get into contact with the cellular glycosylation machinery. *N*-glycosylation patterns differ strongly between organisms and therefore, proteins may end up with non-natural modification patterns. This can reduce or destroy protein function or lead to immune reactions if the proteins are used as pharmaceuticals [14]. Thus, proteins which are sensitive to glycosylation cannot be secreted conventionally in a eukaryotic system without changing the native sequence or adapting the glycosylation machinery of the host [14].

Besides the established canonical secretion pathway, other export mechanisms exist that are characterized by the absence of conventional signal peptides. No common scheme can be deduced for these pathways, rather protein export can be mediated by different mechanisms including various membrane vesicles or by direct translocation via pores or transporters [15]. The diverse phenomena of signal peptide independent secretion are summarized using the term unconventional secretion [16]. The biological functions of unconventional secretion are not well understood. However, it seems that in some cases these pathways are essential to avoid ER/Golgi-based modifications and/or to ensure quality control. The mammalian fibroblast growth factor FGF2 can for example be secreted conventionally, but thereby loses its biological activity [17]. Furthermore, β-galactoside-specific lectins which bind carbohydrates likely avoid conventional secretion to evade potential glycoprotein ligands [18,19].

The fact that ER/Golgi-mediated co- and post-translational modifications are avoided by unconventionally secreted proteins offers new possibilities for applications in biotechnology and medicine [20,21]. On this basis, we have established a novel system for protein production in the corn smut fungus *Ustilago maydis*. Although in its filamentous stage this fungus is the causative agent of corn smut disease, it is safe for biotechnological applications in its non-pathogenic yeast form [22]. Recently, unconventional secretion of the glycoside hydrolase (GH18) family chitinase Cts1 has been demonstrated in *U. maydis* [22,23,24]. While the exact molecular mechanism of unconventional secretion is still under investigation, the pathway has been evaluated for its use in biotechnology over the last few years [24,25,26,27]. Here, heterologous proteins are fused to the N-terminus of Cts1 and are thereby co-exported to the culture supernatant. The bacterial enzyme β-glucuronidase (Gus) has served as a prime example to validate the feasibility of this approach. While Gus is inactivated by *N*-glycosylation during passage of the conventional secretion pathway of eukaryotes, it can be exported in an active state as a Cts1-fusion protein [24,28]. This confirmed that Cts1-mediated unconventional secretion avoids *N*-glycosylation. Naturally, only low amounts of Cts1 are released. However, the use of the protease-deficient strains in combination with a very strong promoter significantly enhanced the yields [25]. Two such strains are available: the first carries a deletion of the preproprotease convertase Kexin 2 (Kex2) that activates secreted proteases, and in the second the genes for five major proteases were deleted sequentially. In these adapted strains other targets like single-chain variable fragments could also be secreted in an active state [21,25].

In this study, we further expanded the list of heterologous targets of the Cts1-based expression system and produced nanobodies derived from camelid heavy-chain antibodies [29]. These molecules of about 15 kDa represent the smallest intact antigen-binding fragments known to date [30,31]. Besides showing expression of an anti-green fluorescent protein (αGfp) nanobody as proof-of-principle, we also for the first time established production of a pharmaceutically relevant protein, namely a nanobody directed against botulinum toxin A (BoNTA).

## 2. Results

### 2.1. Expression and Unconventional Secretion of an αGfp Nanobody

**Figure 1:**
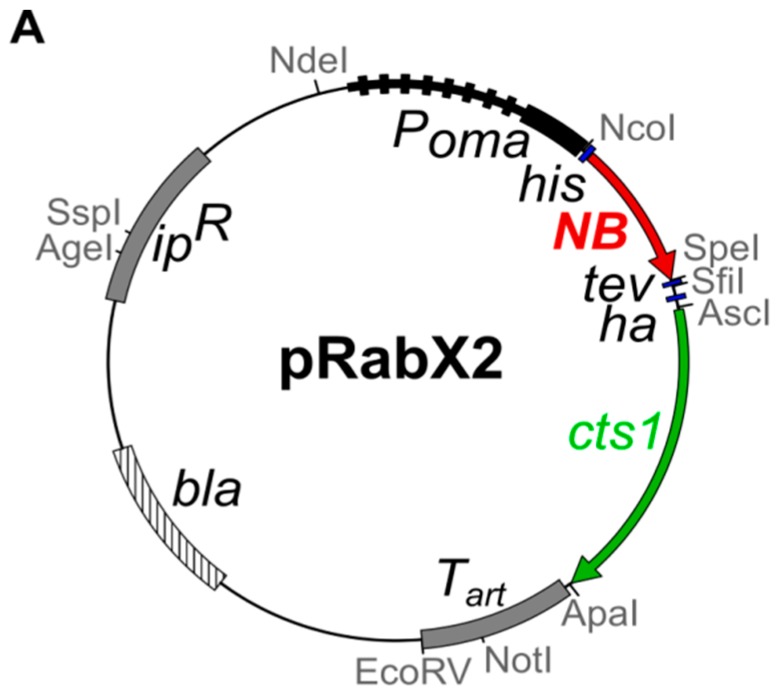

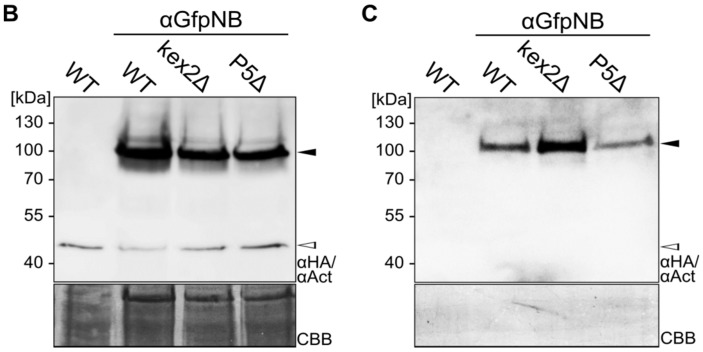
Expression and unconventional secretion of an anti-green fluorescent protein (αGfp) nanobody in *Ustilago maydis*. (**A**) Design of the expression vector for chitinase Cts1-mediated nanobody secretion. Vector backbone pRabX2 carries the *ip^r^* region for targeted homologous recombination. The very strong constitutive promoter P*_oma_* is used for gene expression. The nanobody is expressed as fusion with a sequence encoding Cts1 and tags for purification and detection. A sequence for a *Tobacco Etch Virus* (TEV) protease cleavage site is inserted between the two genes. T*_art_*, artificial terminator composed of the *ubi*1 3′UTR and T*_nos_* [24]. Black arrowhead indicates the full-length fusion protein, open arrowhead depicts the actin loading control; (**B**) Expression of the αGfpNB-Cts1 fusion protein in cell extracts (10 μg) of indicated AB33 (WT) derivatives assayed by Western blot analysis using an αHA antibody. Actin-specific antibodies (αAct) were used in parallel as loading control. The membrane was stained after detection with Coomassie Brilliant Blue (CBB); (**C**) Detection of unconventionally secreted αGfpNB-Cts1 fusion protein in precipitated cell-free culture supernatants (volumetric normalisation) of indicated AB33 (WT) derivatives assayed by Western blot analysis using an αHA antibody. αAct antibodies were used to exclude cell lysis. The membrane was stained with CBB after detection. Black arrowhead indicates the full-length fusion protein, open arrowhead depicts the actin cell lysis control.

To evaluate if functional nanobodies can be produced by Cts1-mediated secretion we chose the green fluorescent protein Gfp as a first antigen. A nanobody directed against Gfp (αGfpNB) has been described in an earlier study [32]. For expression, the respective sequence was adapted to the context-dependent dicodon-usage of *U. maydis* (Table S1). This strategy was chosen because in *U. maydis* premature mRNA polyadenylation has been observed for non-optimized genes [33]. In addition, we prefer dicodon optimization, since not only the codon bias but also the neighbouring nucleotides can influence the choice of this codon from the synonymous group [34]. The optimized gene was inserted into the integrative expression vector pRabX2 (Figure 1A) [25]. In this vector, the gene of interest is translationally fused with the *cts1* gene. The encoded fusion protein harbours an N-terminal Histidin (His) tag for purification and an internal HA tag for detection. In addition, the Cts1 carrier can be removed using an internal *Tobacco Etch Virus* (TEV) protease cleavage site to generate a more natural product with only a small epitope tag (His tag). Gene expression is controlled by the very strong, constitutive, synthetic P*_oma_* promoter [25]. The pRabX2 derivative encoding the αGfpNB-Cts1 fusion was inserted into the *ip* locus of strain AB33 and the two protease deficient strains AB33kex2Δ and AB33P5Δ by homologous recombination [24,25].

To investigate if the αGfpNB-Cts1 fusion protein was produced and secreted, cell extracts and cell-free culture supernatants of the three strains were generated and analysed by Western blot (Figure 1B,C). The 76-kDa protein was present in all cell extracts (Figure 1B). As observed earlier, the Cts1-fusion protein was migrating higher than expected [24]. The amount of extracellular αGfpNB-Cts1 differed depending on the strain background: AB33kex2Δ/αGfpNB-Cts1 showed elevated amounts compared to AB33 αGfpNB-Cts1 and AB33P5Δ/αGfpNB-Cts1 (Figure 1C). While it is not clear why AB33P5Δ/αGfpNB-Cts1 shows a lower protein amount, this was expected for AB33, because this strain still carries all harmful proteases [25]. Although eventually a degradation band was observed which likely accounts to the activity of remaining proteases, AB33kex2Δ/αGfpNB-Cts1 was used for all further experiments, because it contained the highest amount of full-length protein in the culture supernatant (Figure 1C and Figure S1).

### 2.2. Biochemical Characterization of the αGfp Nanobody

**Figure 2:**
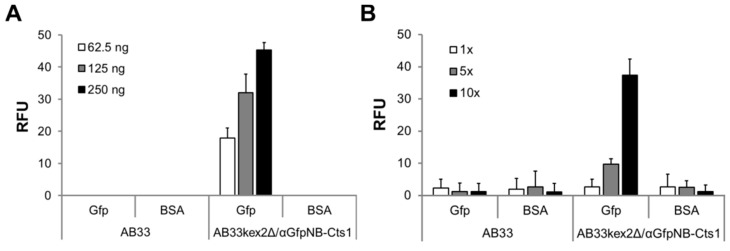
Biochemical characterization of the αGfp nanobody. (**A**) Detection of purified His-tagged green fluorescent protein (Gfp^H^) in enzyme-linked immunosorbent assays (ELISA) using 10 µg of whole cell extracts of strain AB33 (no nanobody) and the Kex2-deficient nanobody expression strain AB33kex2Δ/αGfpNB-Cts1; (**B**) Detection of purified Gfp^H^ in ELISA assays using distinct volumes of immobilized metal affinity chromatography (IMAC)-purified fractions obtained from supernatants of strains AB33 (no nanobody) and AB33kex2Δ/αGfpNB-Cts1. ELISA were performed in biological triplicates. BSA, bovine serum albumin; RFU, relative fluorescence units.

The functionality of the produced nanobody was tested by its binding activity towards the antigen Gfp in enzyme-linked immunosorbent assays (ELISA). To this end, recombinant His-tagged Gfp (Gfp^H^) was produced in *Escherichia coli* using the isopropyl β-d-1-thiogalactopyranoside (IPTG)-inducible pET system (Novagen) and subsequently enriched by immobilized metal affinity chromatography (IMAC; Figure S2). The purified protein was then coupled to ELISA plates and distinct amounts of AB33 lacking the nanobody (negative control) and AB33kex2Δ/αGfpNB-Cts1 (expression strain) cell extracts were added. αHA antibodies were used for detection. Indeed, only wells in which cell extracts of the expression strain containing αGfpNB-Cts1 had been added showed signals which were enhanced with the use of elevated protein amounts (Figure 2A). In addition, we performed a positive control experiment using commercial nanobodies (his-tagged Gfp-binding protein, GfpBP, ChromoTek, Planegg, Germany) with an alternative Gfp antigen (Gfp^S^, Gfp-strep-tag control protein, IBA, Göttingen, Germany; Figure S3). Also, in this experimental setup we detected binding activity for both GfpBP and AB33kex2Δ/αGfpNB-Cts1 cell extracts. Signal saturation occurs at 100 ng of purified GfpBP compared to 10-µg total cell extract, suggesting that as expected αGfpNB-Cts1 constitutes about 1% of the total protein. Altogether, the experiments suggest that the produced nanobody is functional in vitro. The assay was then repeated with purified secreted proteins. Therefore, αGfpNB-Cts1 was enriched from cell-free culture supernatants by IMAC and applied in three different dilutions (Figure 2B). Again, specific signals were obtained only for the proteins purified from the supernatant of the expression strain, confirming secretion of active nanobody.

**Figure 3:**
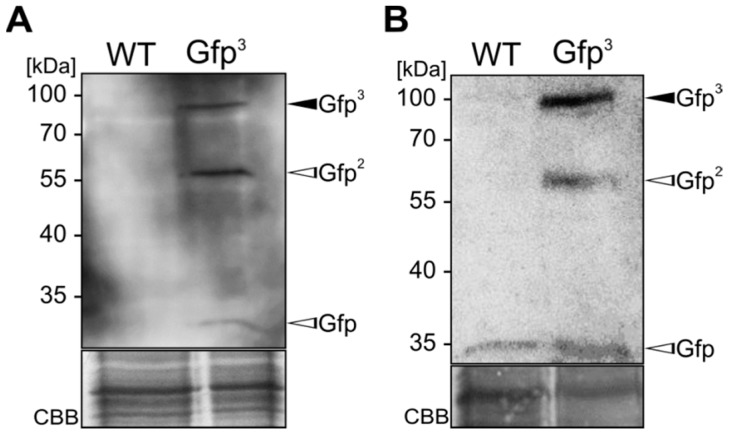
Western blot analyses using an αGfp nanobody produced in *U. maydis*. (**A**) Detection of triple-Gfp (Gfp^3^) by Western blot analysis using the αGfpNB-Cts1 fusion protein enriched from cell extracts of strain AB33kex2Δ/αGfpNB-Cts1; (**B**) Detection of Gfp^3^ by Western blot analysis using αGfpNB-Cts1 enriched from culture supernatants of strain AB33kex2Δ/αGfpNB-Cts1. Besides Gfp^3^ (black arrowheads) its degradation products Gfp^2^ and single Gfp (open arrowheads) were also detected.

As a second proof of nanobody activity we used 10 µg of cell extracts derived from the *U. maydis* strain SG200 [35] and its derivative expressing triple Gfp (SG200 Gfp^3^) [36] for Western blot analysis. The respective membrane was incubated with αGfpNB-Cts1 purified from cell extracts (Figure 3A) or from cell-free supernatants (Figure 3B) of strain AB33kex2Δ/αGfpNB-Cts1. Detection with an αHA antibody revealed signals for triple-Gfp (Gfp^3^) and its degradation products double and single Gfp (Gfp^2^, Gfp) only in the lane containing cells extracts with the antigen, confirming its functionality in antigen binding.

### 2.3. Exploiting Intrinsic Chitin-Binding Properties to Establish a Gfp-Trap

**Figure 4:**
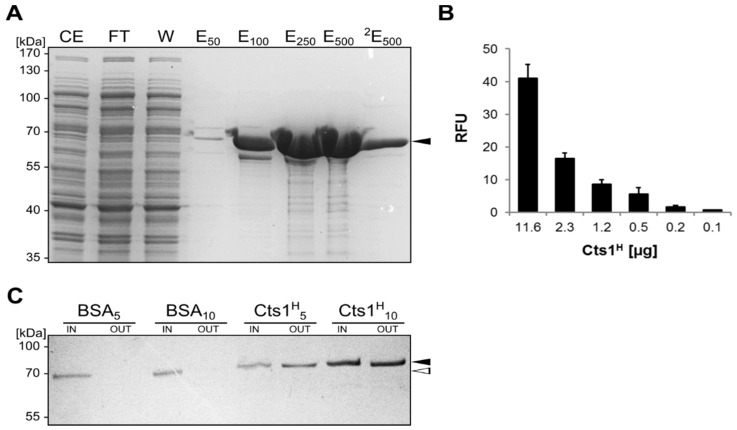
Chitin-binding activity of recombinant Cts1. (**A**) SDS-PAGE analysis of fractions obtained from IMAC purification of His-tagged Cts1 (Cts1^H^) produced in *Escherichia coli*. CE, cell extract; FT, flow through; W, wash step; E, elution fractions using different concentrations of imidazole (subscripts). Cts1^H^ is depicted with a black arrowhead; (**B**) Chitin activity assay with distinct amounts of IMAC purified Cts1^H^. Activity was determined by monitoring the change in fluorescence using the fluorogenic chitinase substrate 4-methylumbelliferyl β-d-*N*,*N*′,*N*′′-triacetylchitotrioside (4-MUC). RFU, relative fluorescence units; (**C**) Chitin-binding activity of distinct amounts (5 or 10 µg) of purified Cts1^H^ (indicated by black arrowhead). Equal amounts of purified BSA (NEB, Ipswich, MA, USA) were used as a negative control (depicted by open arrowhead). Chitin Magnetic Beads were mixed with respective proteins (IN), washed rigorously and bound protein was eluted using Laemmli buffer (OUT).

Chitinases of the GH18 family eventually contain chitin-binding motifs (CBM) for interaction with their substrate [37]. The presence of such domain in Cts1 would provide new potential for biotechnological application because it could be applied for purification of the fusion protein. Bioinformatic predictions did not reveal hints for CBM domains in Cts1 [27]. Nevertheless, we performed in vitro binding assays to test if Cts1 is able to bind chitin. Therefore, we first produced recombinant His-tagged Cts1 in *E. coli*. Using again the Novagen pET system (Merck-Millipore, Darmstadt, Germany), large amounts of soluble His-tagged Cts1 (Cts1^H^) protein could be enriched by IMAC (Figure 4A). The 56-kDa protein again migrated higher in the Sodium dodecyl sulfate (SDS) gel, but Western blot analyses supported the results [38]. In addition, specific chitinase activity assays confirmed that the recombinant protein was biologically active (Figure 4B) [23].

Next, chitin-binding studies were performed using Chitin Magnetic Beads (New England Biolabs, NEB, Ipswich, MA, USA). In contrast to identical amounts of bovine serum albumin (BSA, NEB) used as a control, Cts1 was able to strongly bind to the beads (Figure 4C). This suggests that Cts1 indeed contains a chitin-binding domain that supports the enzyme in its hydrolytic activity towards its substrate. In consequence, a dual use of Cts1 as a secretion vehicle and purification tag was conceivable.

**Figure 5:**
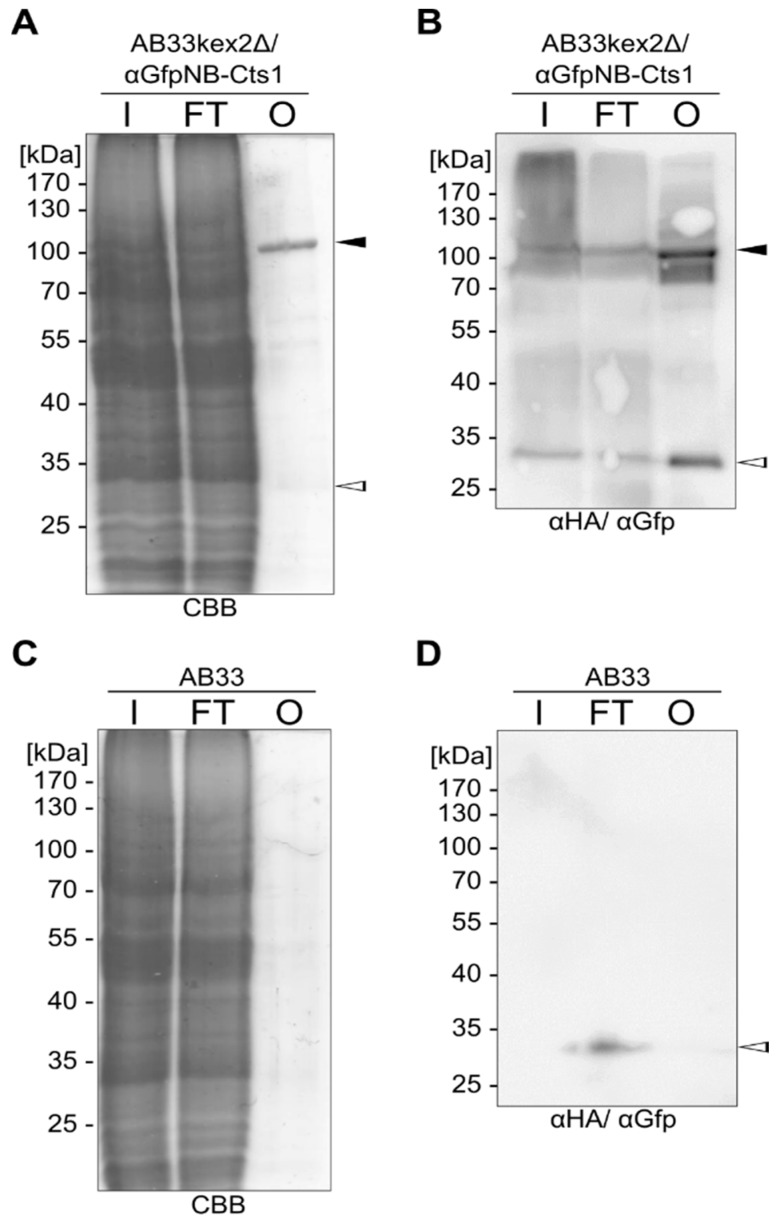
A Gfp-trap based on intrinsic chitin binding activity of Cts1. A total of 1 mg total cell extracts from AB33kexΔ/αGfpNB-Cts1 or AB33 (negative control) were mixed with 0.5 µg purified Gfp^H^ (I, in). After incubation with chitin resin, proteins were eluted with Laemmli buffer (O, out). FT, flow through after incubation of the resin with the protein suspension. (**A**) CBB staining visualizes αGfpNB-Cts1 that had bound to the column (black arrowhead) and a weak band for co-eluted Gfp^H^ (open arrowhead); (**B**) Western blot analyses of the fractions using αHA and αGfp antibodies confirmed the presence of both αGfpNB-Cts1 fusion protein and Gfp^H^ (black and open arrowheads, respectively) in the elution fraction; (**C**) CBB-stained SDS-PAGE and (**D**) Western blot of control experiments in which equally treated AB33 cell extracts were used. Expected running heights of αGfpNB-Cts1 and Gfp^H^ are indicated by black and open arrowheads, respectively. No or strongly reduced Gfp^H^ elution could be observed. Representative results of four biological replicates are depicted.

To apply this finding, we tested if we could generate a Gfp-trap similar to the commercial system (ChromoTek, Planegg-Martinsried, Germany). Therefore, we incubated cell extracts of AB33kexΔ/αGfpNB-Cts1 and AB33 (negative control) with purified Gfp^H^ and incubated the solution with Chitin Resin (NEB, Ipswich, MA, USA). After rigorous washing, proteins were eluted with Laemmli buffer. The αGfpNB-Cts1 fusion protein could be visualized in Coomassie Brilliant Blue (CBB)-stained SDS gels for cell extracts of AB33kexΔ/αGfpNB-Cts1 but not for AB33 purified αGfpNB-Cts1 (Figure 5A,B), showing that chitin affinity is retained in the native protein from *U. maydis* and not hindered by the fusion with the nanobody. In addition, using Western blot analyses, Gfp^H^ could be specifically detected in the elution fraction of the Gfp-trap (using cell extracts of AB33kexΔ/αGfpNB-Cts1; Figure 5B) and not in the control experiment (AB33 cell extracts; Figure 5C,D). Chitin binding activity of Cts1 can thus be exploited for pull-down assays as exemplified by the Gfp-αGfpNB-Cts1 interaction. However, it needs to be mentioned that input protein amounts needed to be carefully balanced for this experiment. In more detail, the amount of input protein needs to be titrated before conducting the actual experiment to exclude unspecific binding. In our experimental setup amounts of up to 500 ng Gfp^H^ could be applied for the assay without detection of unspecifically bound protein in the elution fraction (Figure S4).

### 2.4. Expression, Purification and Biochemical Characterization of a Functional Nanobody against Botulinum Toxin A

**Figure 6:**
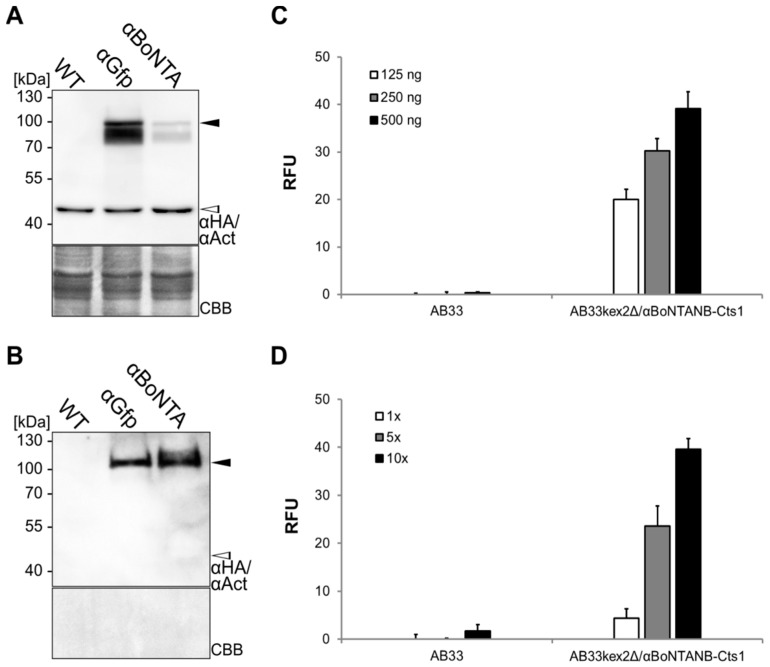
Expression and unconventional secretion of an αBoNTA nanobody. (**A**,**B**) Comparative Western blot analysis of whole cell extracts (**A**) and volumetric precipitated supernatants (**B**) of the nanobody-Cts1 fusion proteins αGfpNB-Cts1 (αGfp) and αBoNTANB-Cts1 (αBoNTA) produced in strain background AB33kex2Δ. The strain AB33kex2Δ (WT) lacking expression constructs was included as negative control. Proteins were detected using an αHA primary antibody. Actin-specific antibodies (αAct) were used as loading control (open arrowhead). The membranes were stained with CBB after detection. Full length protein bands were obtained for both αBoNTANB-Cts1 (black arrowhead, 74 kDa) and αGfpNB-Cts1 (76 kDa); (**C**) Distinct amounts of whole cell extracts derived from AB33 and AB33kex2Δ/αBoNTANB-Cts1 were analyzed by ELISA using botulinum toxin A (BoNTA) as antigen; (**D**) Elution fractions from IMAC purifications of AB33 and AB33kex2Δ/αBoNTANB-Cts1 supernatant were analyzed in different concentrations (10×, 5×, 1×) by ELISA against BoNTA. ELISA were performed in biological triplicates. RFU, relative fluorescence units.

Unconventional secretion was shown to be suited for export of a functional αGfp nanobody. Next, we aimed at testing a pharmaceutically relevant nanobody and decided to produce an αBoNTA nanobody (αBoNTANB) that is able to neutralize the very strong botulinum toxin A (BoNTA) from *Clostridium botulinum* [39]. Expression strain AB33kex2Δ/αBoNTANB-Cts1 was generated as described above, using a pRabX2 derivative containing a sequence encoding the αBoNTA nanobody adapted to the context-dependent codon usage of *U. maydis* (Table S1). Comparative Western blot analyses confirmed the presence of the fusion protein αBoNTANB-Cts1 in cell extracts (Figure 6A) and precipitated supernatants (Figure 6B) indicating that the protein is produced and secreted. Of note, the amount of αBoNTANB-Cts1 in culture supernatants was greater compared to αGfpNB-Cts1 (Figure 6B). Antigen-binding activity was then assayed using commercial BoNTA-coated ELISA plates (Metabiologics, Inc., Madison, WI, USA). Binding activity could be confirmed both in whole cell extracts and protein enriched from culture supernatants by IMAC (Figure 6C,D). This demonstrates that unconventional secretion can be exploited to generate pharmacologically relevant antibody formats.

### 2.5. Optimizing αBoNTA Nanobody Expression

**Figure 7:**
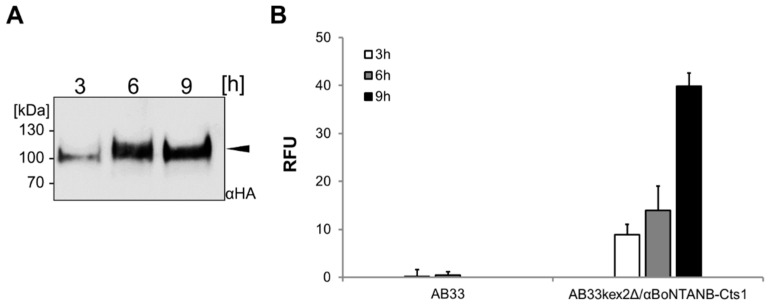
Optimization of αBoNTA nanobody expression. (**A**) Precipitated cell-free culture supernatants after different times of incubation. The full length fusion protein is depicted with a black arrowhead. Identical volumes of a single culture were analyzed; (**B**) Supernatant samples harvested at indicated time points were subjected to ELISA against BoNTA (biological triplicates). RFU, relative fluorescence units.

For initial experiments and biochemical characterization, nanobody expression cultures were harvested at low optical densities (0.5–1). This yielded low levels of secreted protein. To characterize the behavior of cultures grown to higher optical densities, we incubated the expression strain AB33kex2Δ/αBoNTANB-Cts1 for 9 h in shake flask batch cultures, corresponding to a final optical density of about 3.0. Western blot analyses using volumetric samples taken at different time-points now showed increasingly strong bands for the full length protein (Figure 7A). In line with that, ELISA assays demonstrated increasing signals of the samples harvested over time (Figure 7B). To determine the approximate amount of αBoNTANB-Cts1 secreted in the batch culture, quantitative Western blot analyses were performed using a dilution series of commercial MultiTag^®^ Protein (GenScript, Piscataway, NJ, USA) as an internal standard (Figure S5). According to this quantification, a yield of about 140 µg/L was achieved in the standard batch culture.

## 3. Discussion

In this study we produced two different heavy-chain antibody-derived nanobodies by Cts1-mediated unconventional secretion in the yeast stage of the fungus *U. maydis*. As a proof-of-principle we first tested a nanobody against Gfp. This nanobody was initially described by Rothbauer and colleagues and was expressed intracellularly as a chromobody in mammalian cells [32]. The group also invented a first versatile nanotrap that was later on developed further and commercialized by the company ChromoTek [40]. Here, we achieved the unconventional secretion of the αGfpNB in its active form in vitro, suggesting that nanobodies are a suitable target for Cts1-mediated export. In addition, we made the important observation that although Cts1 lacks a classical chitin-binding motif it still sticks to chitin in both its native state or after recombinant expression in *E. coli*. While chitin binding activity was clearly detected, we did not observe any cleavage of the polysaccharide in in vitro assays [38]. Therefore, this fact can be used to purify Cts1 including its N-terminal protein fusions. In addition, similarly to the commercial Gfp nanotrap, we showed that coupling of αGfpNB-Cts1 to chitin can be applied to pull-down Gfp—now exploiting an intrinsic feature of the carrier Cts1. Importantly, with careful design and appropriate controls this experiment can now be expanded to other applications, for example to perform pull-down experiments with potential Cts1 interaction partners or they may even be applied to identify those. The fact that Cts1 protein produced in *E. coli* is enzymatically active furthermore confirms that it does not need eukaryotic post-translational modifications for function. This is in line with its unconventional secretion during cell separation [27].

In addition, we expressed a camelid-derived nanobody directed against BoNTA [39]. This is the first example of products of pharmaceutical relevance generated by unconventional secretion in *U. maydis*. The functionality of the nanobody in binding its cognate antigen BoNTA in the culture supernatant indicates that the protein folds correctly. However, to finally verify its biological function, studies on BoNTA neutralization would need to be performed in an in vivo model as has been shown in the original study for a mouse or a primary neuron botulism model [38]. To this end, it would be important to also express nanobodies with other neutralizing epitopes such as the αE-Tag in order to expand the clearing potential of the antitoxin and thus enhance the neutralizing effect in vivo [39]. In the future, such nanobodies could deal as an alternative to polyclonal antisera used to date for treating botulism in humans [41,42]. In this therapy it is crucial that the antitoxin both neutralizes toxin function and promotes clearance of toxin from the body which has already been demonstrated for the nanobodies after intravenous injection in a mouse model [39].

Of note, in our study the produced nanobodies carry a small N-terminal His-tag as well as the large C-terminal fusion to Cts1. Tags can potentially interfere with protein function e.g., by blocking the antigen binding site. However, we consider it unlikely that these tags influence the binding activity of the nanobodies because similar N- and C-terminal extensions have been used successfully in other studies [43,44,45,46]. Furthermore, as estimated by the comparison to the positive control GfpBP, the produced αGfpNB-Cts1 nanobody fusion shows the expected activity in cell extracts. However, to completely exclude potential steric interferences, we plan to remove the Cts1 moiety by protease cleavage in the future.

The recombinant expression of nanobodies has been described in diverse studies using for example mammalian cell lines, plants and different microorganisms including *Saccharomyces cerevisiae* and *Aspergillus oryzae* with yields in the mg/L range [47,48,49]. Hence, this antibody format is not the most difficult to produce [47]. Therefore, in the next step we will focus on proteinaceous biopharmaceuticals which are more challenging to obtain in microbial systems and try to establish their expression in *U. maydis*. An interesting target could for instance be the plasmodial surface associated protein P*f*RH5 which has been shown to be a highly potent vaccine target against malaria [50,51]. In general, proteins of plasmodial origin are difficult to produce in the most commonly available expression systems such as yeasts or bacteria [52,53,54,55]. Interestingly, it has been shown that *N*-linked glycosylation impairs functionality of P*f*RH5, making it a perfect target for unconventional secretion in *U. maydis* [56,57].

Still, with about 140 µg/L the yields obtained in our secretory system are too low to be competitive. However, we believe that with different optimization steps we will evolve Cts1-mediated unconventional secretion into a relevant alternative platform for the production of high-value proteins. Even using protease-deficient strains as expression hosts we observe significant degradation of our heterologous target proteins, which limits the yield of active full length protein (unpublished observation) and may even be disruptive during application. Hence, we are currently identifying and eliminating more involved harmful proteases. In addition, medium optimization combined with upscaling to the bioreactor can improve yields to a relevant range. It has been shown before that switching from shake-flask to fermentation can result in yield increase up to 10-fold [58]. Lastly, solving the exact molecular mechanism of unconventional Cts1 secretion will provide novel tools to increase its secretion. Factors essential for its export could for example be overexpressed to boost the pathway. Also, the identification of other proteins following a similar pathway could provide valuable mechanistic insights.

In summary, our study contributes to the rising field of eukaryotic unconventional secretion covered in this special issue and demonstrates its potential for applied research. Our expression system opens up new possibilities for the production of valuable non-glycosylated proteins in an inexpensive eukaryotic system. Target proteins could even originate from prokaryotic sources since these naturally lack glycosylation and thus, their proteins are often sensitive towards this modification. In bacteria the signal sequence independent secretion has recently been recognized as a potential alternative way to export heterologous proteins which overburden the conventional Sec or Tat pathways [59,60]. Our study may now provide the starting point to inspire the broader application of other specialized eukaryotic unconventional secretion pathways for applied research in the near future. With the production of several different heterologous proteins like Gus, single-chain variable fragments (scFvs) or nanobodies [24,25] (this study), we have now laid a solid foundation for follow-up studies concentrating on other relevant targets that fulfill the criteria for unconventional secretion and are hard to produce in established systems.

## 4. Materials and Methods

### 4.1. Microbial Strains, Culture Conditions and Plasmids

The *E. coli* K-12 derivate Top10 (Invitrogen/Life Technologies) was used as a host for molecular cloning. *E. coli* Rosetta 2 (DE3) pLysS (Merck-Millipore, Darmstadt, Germany; Table 1) was employed for protein expression (see Section 4.5). Bacterial expression cultures were grown at 37 °C with 200 rpm shaking.

*U. maydis* strains used in this study are listed in Table 2. Cultures were grown in complete medium [61] supplemented with 1% (*w*/*v*) glucose (CM-Glc) at 28 °C with 200 rpm shaking using baffled flasks. An optical density of 1 (*λ* = 600 nm) relates to about 1–2 × 10^7^ cells/mL [62].

*U. maydis* strains used in this study are listed in Table 2. All newly generated mutants were obtained by transformation of the progenitor strains with linearized plasmids. For this purpose, integrative expression plasmids derived from pRabX2 [25] (Table 2) were used which contain a region encoding an *ip* allele that confers resistance to the antibiotic carboxin (*ip^r^*). For integration into the *ip^s^* locus by homologous recombination, respective plasmids were linearized within the *ip^r^* gene [24]. Subsequently, protoplasts were transformed with the linearized plasmids following published methods [63]. Homologous single or multiple integrations at the *ip* locus were verified by Southern blot analysis using a 2.1-kb probe obtained with the primer combination oMF502 × oMF503 (Table 3) and the template pUMa260 [63,64].

To generate plasmid pRabX2 P_oma_His-αGfpNB-TH-Cts1 (pUMa2240) the sequence of the llama-derived αGfp nanobody [32] was dicodon-optimized (Table S1) and synthetized by the company GeneArt (Thermo Fisher) yielding vector pMA-T Um-anti-Gfp-NB (pUMa2234). The 403-bp coding sequence was next amplified from this template using oRL1577 and oRL1578 introducing terminal *Nco*I and *Spe*I sites. The PCR product was hydrolyzed with *Nco*I and *Spe*I and inserted into the respective sites of pUMa2137 [25] replacing the *scFv* gene.

Dicodon-optimized αBoNTA (Table S2) derived from camelids (ciA-H7, gene bank acc. HQ700708) [38] was synthetized by IDT (Belgium) and delivered in vector pUCIDT_UmciA-H7 (pUMa2858). pRabX2 P_oma_His-αBoNTANB-TH-Cts1 (pUMa2863) was subsequently obtained using a 371-bp fragment, containing the dicodon-optimized sequence for a His-UmciA-H7 NB (αBoNTA) fusion protein from pUMa2858, which was cloned into the pUMa2240 expression vector backbone [25], using the restriction endonucleases *Nco*I and *Spe*I.

For generation of pET15b_His-Cts1 (pUMa1951) the *cts1* gene was amplified by PCR on the template pUMa1521 [25] using primers oRL1085 and oRL1086. The resulting PCR product (1524 bp) was hydrolyzed with *Nde*I and *Bam*HI and inserted into the respective sites of the pET15b vector (Novagen/Merck-Millipore). For generation of pET15b_His-Gfp (pUMa2156) the *gfp* gene was amplified from pUMa828 [23] using primers oRL1384 and oRL1385. The 722-bp product was hydrolysed with *Nde*I and *Bam*HI and inserted into the backbone of pUMa1951 (this study) using the same enzymes, thereby replacing the *cts1* open reading frame.

### 4.2. Purification of Cts1-Fusion Proteins from U. maydis

Proteins were purified via their N-terminal 10× Histidin (His) tag from native cell extracts or cell-free culture supernatants using Ni^2+^-nitrilotriacetic acid (NTA) agarose (Protino^®^, Macherey Nagel, Düren, Germany). The preparation of cell extracts and precipitation of proteins from cell-free culture supernatants have been described before [24,28]. Purification was performed according to the Qiagen Expressionist manual. Therefore, 1 mL of the matrix was equilibrated with 10 mL lysis buffer (50 mM NaH_2_PO_4_, 300 mM NaCl, 10 mM imidazole, pH 8.0). A total of 1.5 mL of native cell extracts was incubated with the equilibrated matrix in a batch procedure (1 h, 4 °C). For protein purification from supernatants, a 200 to 500 mL culture was grown to an OD_600_ of 0.75, centrifuged (7000 rpm, 10 min, 4 °C) and the supernatant subsequently filtered (MN 615^1/4^, 150 mm, Macherey Nagel). After addition of one cOmplete™ EDTA-free Protease Inhibitor Cocktail tablet (Roche, Basel, Switzerland) per 200 mL supernatant, it was incubated with the equilibrated Ni^2+^-NTA matrix in a batch procedure (1 h, 4 °C). In both cases, after incubation, the matrix was transferred into empty purification columns and washed with 2 mL of lysis buffer containing different concentrations of imidazole (50 mM NaH_2_PO_4_, 300 mM NaCl supplemented with 20, 50 and 100 mM imidazole, pH 8.0). For elution, the matrix was first treated with 1 mL lysis buffer containing 250 mM imidazole and then with 2 mL containing 500 mM imidazole. Samples of all fractions were collected and analyzed by SDS-PAGE. All steps were performed at 4 °C.

### 4.3. SDS-PAGE and Western Blot Analysis

For SDS-PAGE analysis, 10% acrylamide gels were used. Prior to loading, denaturing 3× Laemmli sample buffer [66] was added to all samples, followed by boiling at 98 °C for 10 min and subsequent centrifugation at 15,000× *g* at room temperature for 2 min. Gels were either stained with Coomassie Brilliant Blue (CBB) or blotted onto polyvinylidene difluoride (PVDF) membranes. TBS-T (20 mM Tris-HCl pH 7.6, 136 mM NaCl, 0.05% (*v*/*v*) Tween-20) supplemented with 3% (*v*/*v*) skimmed milk was applied for blocking. A mouse αHA antibody (Roche) was used as primary antibody in a dilution of 1:4000. When indicated, a mouse antibody directed against actin (αAct; MP Biomedicals, Singapore) was in parallel applied in a dilution of 1:1500. A horse αmouse antibody conjugated to horseradish peroxidase (Promega, Madison, WI, USA) dealt as secondary antibody in a concentration of 1:10,000. After incubating the membrane for 1 min in horseradish peroxidase (HRP)-substrate solution (AceGlow™, VWR, Erlangen, Germany), chemiluminescence was detected using the LAS4000 (GE Healthcare, Little Chalfont, UK).

CBB staining was used to stain polyacrylamide gels and PVDF membranes. Gels were incubated in Coomassie staining solution (0.05% Coomassie Brilliant Blue R250, 15% (*v*/*v*) acetic acid, 15% (*v*/*v*) methanol) for 1 h on an orbital shaker and then washed with H_2_O. After incubation for at least 4 h in destaining solution (15% (*v*/*v*) acetic acid, 15% (*v*/*v*) methanol), the gel was washed for 1 h in H_2_O. PVDF membranes were incubated for 20 min in Coomassie staining solution and 20 min in destaining solution with a subsequent 10-min H_2_O washing step after chemiluminescence detection. The membranes were then dried before documentation to reduce background staining.

For quantitative Western blot analysis, commercial purified MultiTag^®^ Protein (LifeTein, Somerset, NJ, USA) was used in defined amounts. Signals were quantified with the Image Studio Lite software (Version 5.2.2, LI-COR, Lincoln, NB, USA).

To analyze the binding activity of the αGfpNB isolated from *U. maydis* cell extracts, a modified Western blot protocol was applied. First 10 μg of native cell extracts from a 3xGfp-expressing strain (UMa587) and of a wildtype negative control (UMa67) were subjected to SDS-PAGE und subsequently blotted on a PVDF membrane. The membrane was then blocked with 3% (*v*/*v*) skimmed milk in PBST (137 mM NaCl, 2.7 mM KCl, 10 mM Na_2_HPO_4_, 1.8 mM KH_2_PO_4_, pH 7.4, 0.05% (*v*/*v*) Tween-20). To specifically detect the Gfp on the PVDF membrane, IMAC-purified αGfpNB-Cts1 fusion protein from UMa1397 cell extracts were used as primary antibodies. Identical volumes of purified protein obtained from UMa1391 were used as negative control. Membranes were incubated overnight on 4 °C with the purified nanobody mixed with blocking buffer (PBST with 3% (*v*/*v*) skimmed milk). After washing three times with PBST, the standard Western blot detection protocol was followed using αHA (1:4000, Roche) and horse αmouse-HRP antibodies (1:10,000, Promega).

### 4.4. Enzyme-Linked Immunosorbent Assay (ELISA)

For detection of αGfp binding activity, protein-adsorbing 96-well microtiter plates (Nunc MaxiSorp^®^, ThermoFisher Scientific, Waltham, MA, USA) wells were coated with 2 µg Gfp^H^ (purified from *E. coli*, see Section 4.5), 2 µg BSA (NEB, Ipswich, MA, USA; negative control) or 2 µg Gfp^S^ (Gfp-strep-tag, IBA, Göttingen, Germany; positive control) in 100 mM bicarbonate coating buffer (100 mM HCO_3_) at 4 °C overnight. Blocking was conducted for at least 4 h at room temperature with 4% (*w*/*v*) skimmed milk in PBS (137 mM NaCl, 2.7 mM KCl, 10 mM Na_2_HPO_4_, 1.8 mM KH_2_PO_4_, pH 7.4) and subsequently wells were washed three times with PBST (PBS supplemented with 0.05% (*v*/*v*) Tween-20). αGfpNB-Cts1 containing samples and appropriate controls were supplemented with skimmed milk in PBS (4% *w*/*v* final concentration, f.c.) and then applied either in defined volumes or protein amounts. The plate was incubated with the samples and controls overnight at 4 °C. After 3× PBS-T washing, a mouse αHA primary antibody 1:4000 diluted in PBS supplemented with skimmed milk (4% *w*/*v* f.c.) was added and incubated for 2 h at room temperature. Alternatively, for the positive control, Gfp-binding protein (ChromoTek) or indicated cell extracts were added and detected with a mouse αHis antibody (1:5000, Sigma-Aldrich, Saint Louis, MO, USA). Then, wells were washed again three times with PBS-T and incubated with a horse αmouse-HRP secondary antibody for 1 h at room temperature (1:5000 in PBS supplemented with skimmed milk (4% *w*/*v* f.c.)). After washing again three times with PBST and three times with PBS, 100 µL QuantaRed™ Enhanced Chemifluorescent HRP substrate (ThermoFisher Scientific, Waltham, MA, USA) per well were added. After incubation at room temperature for 1 h, the reaction was stopped with 10 µL of stop solution (kit component) and the reactions were transferred into Black µClear^®^ 96 Chimney Well plates (flat bottom, Cellstar^®^; Greiner Bio-One, Kremsmünster, Austria). Fluorescence readout was performed at 570 nm (excitation) and 600 nm (emission) using an Infinite M200 plate reader (Tecan, Männedorf, Switzerland).

To test antigen binding of αBoNTANB-Cts1, BoNTA-coated 96-well microtiter plates (Metabiologics Inc., Madison, WI, USA) were used. The wells were blocked with 4% (*w*/*v*) skimmed milk in PBS for at least 4 h at room temperature and after 3× PBS-T washing, αBoNTANB-Cts1 containing cell extracts, supernatants or purified αBoNTANB-Cts1 from cell extracts or supernatants as well as the corresponding negative controls were applied to the wells either in defined volumes or protein concentrations. Subsequently, the protocol described above was followed.

### 4.5. E. coli Expression and Purification Gfp and Chitinase

Heterologous expression of Gfp^H^ and Cts1^H^ (both proteins tagged with a 6× His tag at the N-terminus) was performed in *E. coli* Rosetta (DE3) pLysS using plasmids pET15b_His-Gfp (pUMa2156) and pET15b_His-Cts1 (pUMa1951), respectively. For expression of Cts1^H^ and Gfp^H^ transformants of *E. coli* Rosetta (DE3), pLysS harboring the respective expression plasmid were grown at 37 °C to an OD_600_ of 0.6. Then, expression was induced with 0.5 mM IPTG and the culture was incubated for further 3 h until harvest. The cell pellet was resuspended in lysis buffer (50 mM NaH_2_PO_4_, 300 mM NaCl, 10 mM imidazole, supplemented with cOmplete^®^ EDTA-free Protease Inhibitor Cocktail (1 tablet/100 mL; Roche)). Cells were disrupted using an ultrasonic sonotrode (5 mm microtip (Heinemann, Portsmouth, NH, USA)) attached to the Cell Disruptor B15 device (Branson; stage 4, 3 × 30 s pulse, 3 repetitions). Cell extracts were subsequently centrifuged at 5000× *g* for 10 min (4 °C) and subjected to purification. Soluble proteins were purified at 4 °C with immobilized metal affinity chromatography (IMAC) following standard protocols (The QIAexpressionist, Qiagen, Hilden, Germany). To enhance binding, the protein extracts were batch incubated with Ni^2+^-NTA matrix for 1 h at 4 °C with gentle agitation. After collecting the flow through, the matrix was washed with 10 and 20 mM imidazole. Elution occurred stepwise using up to 250 mM imidazole (Gfp^H^) or 500 mM imidazole (Cts1^H^) in lysis buffer. Subsequently, a buffer exchange was conducted with different elution fractions enriched in Cts1^H^ or Gfp^H^ using PD10-columns (GE Healthcare) following the manufacturer’s protocol. Purified target protein was then eluted in potassium-hepes-magnesium chloride (KHM) buffer (Cts1^H^; 110 mM potassium acetate, 20 mM HEPES, 2 mM MgCl_2_) and supplemented with 10% (*v*/*v*) glycerol or PBS/glycerol (Gfp^H^; PBS supplemented with 10% (*v*/*v*) glycerol). Protein concentration was determined using Bio-Rad Bradford Protein Assay (Bio-Rad Laboratories, Hercules, CA, USA; Bradford, 1976). All obtained fractions were analyzed using SDS-PAGE and Western blot analysis. Eluted proteins were stored at −20 °C until further use.

### 4.6. Chitin Binding Assay

Chitin binding activity of Cts1^H^ purified from *E. coli* was assayed using chitin-coated magnetic beads (NEB, Ipswich, MA, USA). First, 50 µL magnetic bead slurry was washed twice with 500 µL chitin binding buffer (CBD, 500 mM NaCl, 20 mM Tris-HCl, 1 mM EDTA, 0.1% (*v*/*v*) Tween-20, pH 8.0). The supernatant was discarded. Next, 5 or 10 µg purified Cts1^H^ and commercial purified bovine serum albumin (BSA, NEB, Ipswich, MA, USA), respectively, were incubated in a total volume of 500 µL CBD buffer with the washed beads for 1 h at 4 °C with agitation. Subsequently, the supernatant was discarded and unbound protein was washed off with 500 µL CBD buffer for 30 min with agitation (4 °C). The step was repeated once. Finally, three short washing steps with 500 µL CBD buffer without incubation but with multiple inversions of the tube were conducted. To elute bound protein, 15 µL 1× Laemmli buffer were added to the beads and incubated for 5 min at room temperature. The supernatant was transferred to a new tube and boiled for 5 min prior to SDS-PAGE analysis.

### 4.7. Chitinase Activity Assay

Chitinolytic activity of IMAC purified Cts1^H^ was analyzed using the fluorogenic chitinase substrate 4-Methylumbelliferyl-β-d-*N*,*N*′,*N*′′-triacetylchitotriosid (4-MUC; Sigma-Aldrich, Taufkirchen, Germany). To this end, a 200 ng/mL 4-MUC stock solution was prepared in KHM buffer (110 mM CH_3_CO_2_K, 20 mM HEPES (pH 7.3), 2 nm MgCl_2_). A quantity of 70 µL of the substrate solution was mixed with 30 µL sample solution (11.6, 2.3, 1.2, 0.5, 0.2, 0.1 µg in H_2_O_bid_) in Black µClear^®^ 96 Chimney Well plates (flat bottom, Cellstar^®^; Greiner Bio-One). The plate was immediately covered with Parafilm^®^ M (Brand^®^, Wertheim, Germany), protected from light and incubated at 37 °C for one hour. The enzymatic reaction was stopped by adding 200 µL 1 M Na_2_CO_3_ and fluorescence measurements were performed at 360 nm (excitation) and 450 nm (emission) in an Infinite^®^ 200 PRO plate reader (Tecan Group AG, Männedorf, Switzerland). Fluorescence of each sample was determined in technical triplicates.

### 4.8. Gfp Pull-Down with Chitin-Bound Cts1

αGfpNB-Cts1 full-length protein was enriched from native cell extracts of AB33kex2Δ/αGfpNB-Cts1 using chitin resin. For the cell extraction cells of AB33kex2Δ/αGfpNB-Cts1 or AB33 (negative control without αGfpNB-Cts1) were harvested at their logarithmical growth phase (OD_600_ = 0.7). Pellets were resuspended in 2 mL native extraction buffer (chitin binding buffer; 500 mM NaCl, 50 mM Tris-HCl (pH 8), 0.5 mM EDTA, 0.1% (*v*/*v*) Tween-20, 1 mM PMSF, 2.5 mM benzamidine, and 2× cOmplete™ EDTA-free Protease Inhibitor Cocktail (Roche)) and frozen in liquid nitrogen. Cell extraction was performed using a pebble mill (Retsch; 5 min at 30 Hz). After centrifugation (6000× *g* for 30 min at 4 °C) the protein concentration was determined by Bradford Protein Assay (Bio-Rad; Bradford, 1976). A total of 1 mg of total protein was supplemented with 100 µL of Chitin Resin (NEB, Ipswich, MA, USA, S6651L) and 0.5 µg of Gfp^H^ (expressed and IMAC purified from *E. coli* (see Section 4.5)). Alternatively, for the titration of Gfp^H^ loading, the experiment was performed without cell extracts using the indicated amounts of purified Gfp^H^. The suspension was incubated for 3 h at 4 °C with agitation. Centrifugation chromatography columns (Pierce^®^ Spin Cups, Thermo Scientific, Waltham, MA, USA) were loaded with the suspension. After collection of the flow through fraction (0.9× *g* for 1 min at 4 °C) the resin was subjected to four washing steps with each 400 µL chitin binding buffer. All collected fractions were treated with trichloroacetic acid (TCA) to precipitate contained proteins. Finally, 20 µL of 3× Laemmli buffer (150 mM Tris-HCl (pH 6.8), 6% (*v*/*v*) SDS, 30% (*v*/*v*) glycerol, 15% (*w*/*v*) β-mercaptoethanol, 0.003% (*w*/*v*) bromophenol blue) were added to the column and incubated at 95 °C for 5 min to elute αGfpNB-Cts1 (0.9× *g* for 2 min). To analyze enrichment of αGfpNB-Cts1 and Gfp^H^, pull-down precipitated fractions were subjected to SDS-PAGE and Western blot analysis.

## 5. Conclusions

The constantly rising request for recombinant proteins in both fundamental and applied research calls for the establishment of alternative expression systems that complement existing platforms. High-value proteins are of special interest for the field of red biotechnology where these products are applied in medicine and diagnostics. In the present study we used the previously described Cts1-mediated unconventional secretion pathway in *U. maydis* to co-export functional nanobodies directed against botulinum toxin A. This is the first example for a pharmaceutically relevant protein produced via this pathway in *U. maydis*. Currently, we are optimizing the expression system on different levels in order to obtain higher yields and less degradation of the exported proteins in the culture broth. Hence, in the future the optimized system will provide a valuable tool to produce otherwise difficult-to-express proteinaceous biopharmaceuticals in an easy to handle and innocuous eukaryotic host.

## Figures and Tables

**Table 1:** *E. coli* strains used for protein expression.

Strain	Relevant Genotype/Resistance	Uma ^1^	Plasmid Transformed	Progenitor	Reference
*E. coli* Rosetta2 (DE3) pLysS	*F*^−^ *ompT hsdS_B_* (r_B_^−^ m_B_^−^) *gal dcm* (DE3) pLysSRARE2 (CamR)	791	-	-	Novagen (Merck-Millipore)
*E. coli* Rosetta2 (DE3) pLysS pET15b_His-Gfp	*F*^−^ *ompT hsdS_B_* (r_B_^−^ m_B_^−^) *gal dcm* pLysSRARE2 (CamR) pET15b_His-Gfp (AmpR)	1464	pET15b_His-Gfp (pUMa2156) (expression of a 6xHis-Gfp fusion protein)	UMa791	this study
*E. coli* Rosetta2 (DE3) pLysS pET15b_His-Cts1	*F*^−^ *ompT hsdS_B_* (r_B_^−^ m_B_^−^) *gal dcm* pLysSRARE2 (CamR) pET15b_His-Cts1 (AmpR)	1170	pET15b_His-Cts1 (pUMa1951) (expression of a 6xHis-Cts1 fusion protein)	UMa791	this study

^1^ Internal strain collection number.

**Table 2:** *U. maydis* strains used in this study.

Strains	Relevant Genotype/Resistance	UMa ^1^	Reference	Plasmid Integrated	Manipulated Locus	Progenitor Strain
AB33	*a2 P_nar_bW2bE1*	133	[65]	-	*b*	FB2
	*PhleoR*					
AB33 αGfp-Cts1	*ip^r^[P_oma_his:agpf:tev:hacts1:ubi1 3′UTR] ip^s^*	1396	this study	pRabX2 PomaHis-αGfp-NB-TH-Cts1 (pUMa2240)	*cbx*	AB33
	*CbxR*					
AB33kex2Δ/αGfp-Cts1	*FRTwt[um02843Δ::hyg]FRTwt*	1397	this study	pRabX2 PomaHis-αGfp-NB-TH-Cts1 (pUMa2240)	*cbx*	UMa803 [25]
	*ip^r^[P_oma_his:agpf:tev:ha:cts1:ubi1 3′UTR] ip^s^*					
	*CbxR*, *HygR*					
AB33P5Δ/αGfp-Cts1	*FRT5[um04400Δ::hyg]FRT5*	1465	this study	pRabX2 PomaHis-αGfp-NB-TH-Cts1 (pUMa2240)	*cbx*	UMa1391 [25]
	*FRT3[um11908Δ]*					
	*FRT2[um00064 Δ]*					
	*FRTwt[um02178Δ]*					
	*FRT1[um04926Δ]*					
	*ip^r^[P_oma_his:agpf:tev:ha:cts1:ubi1 3′UTR] ip^s^*					
	*PhleoR*, *CbxR*					
SG200	*a1:mfa2*, *bE1*, *bW2*	67	[35]	-	-	-
	*PhleoR*					
SG200 Gfp^3^	*ip^r^[P_otef_egfp:egfp:egfp]ip^s^*	587	[36]	-	*cbx*	SG200 [35]
	*CbxR*					
AB33kex2Δ/αBoNTA-Cts1	*FRTwt[um02843Δ::hyg]*	1870	this study	pRabX2 PomaHis-αBoNTA-NB-TH-Cts1 (pUMa2863)	*cbx*	UMa803 [25]
	*FRTwt ip^r^[P_oma_his:abonta:tev:ha:cts1:ubi1 3′UTR] ip^s^*					
	*PhleoR*, *HygR*, *CbxR*					

^1^ Internal strain collection number.

**Table 3:** DNA oligonucleotides used in this study.

Designation	Nucleotide Sequence (5′–3′)
oMF502	ACGACGTTGTAAAACGACGGCCAG
oMF503	TTCACACAGGAAACAGCTATGACC
oRL1085	CACCATATGTTTGGACGTCTTAAGCACAGGATGTCTCGCGCTCGACTAGACG
oRL1086	GTGGGATCCTTACTTGAGGCCGTTCTTGACATTGTCCC
oRL1384	GTGGGATCCTTACTTGTACAGCTCGTCCATGCCG
oRL1385	CACCATATGGTGAGCAAGGGCGAGGAGC
oRL1577	GCCATGGCGGCCCATCACCACCATCACCACCATCACCACCATCATATGGCCGACGTCCAGCT
oRL1578	GACTAGTCGACGAGACGGTGA

## Supplementary Materials

Supplementary materials can be found at www.mdpi.com/1422-0067/18/5/937/s1.

## Abbreviations

ER	Endoplasmic reticulum
NB	Nanobody; BoNTA, botulinum toxin A
α	Anti
CBB	Coomassie brilliant blue
Gfp	Green fluorescent protein
IMAC	Immobilized metal affinity chromatography
