# Supplementary material for: Applying Unconventional Secretion in Ustilago maydis for the Export of Functional Nanobodies
Source: Int J Mol Sci. 2017 Apr 29;18(5):937. doi: 10.3390/ijms18050937 (PMC5454850; doi:10.3390/ijms18050937)
Supplement: Supplementary file 1 [file ijms-18-00937-s001.pdf]

Supplementary Materials: Applying Unconventional Secretion in *Ustilago maydis* for the Export of Functional Nanobodies

Marius Terfrüchte, Michèle Reindl, Silke Jankowski, Parveen Sarkari, Michael Feldbrügge and Kerstin Schipper

Table S1. Sequences of genes optimized according to the context-dependent dicodon usage of *Ustilago maydis* (*U. maydis*).

Encoded protein	Codon-optimized gene sequence (5′–3′)	Company
anti-Gfp nanobody (αGfpNB) [1]	ATGGCCGACGTCCAGCTCGTCGAGTCGGGTGGTGCGCTCG<br>TCCAGCCCGGTGGCTCGCTGCGTCTCTCGTGCGCCGCCTCG<br>GGCTTCCCCGTCAACCGCTACTCGATGCGATGGTACCGTC<br>AGGCGCCTGGCAAGGAGCGCGAGTGGGTCGCCGGCATGT<br>CGTCGGCCGGTGACCGATCGTCGTACGAGGACTCGGTCAA<br>GGGTCGCTTCACCATCTCGCGCGACGACGCTCGCAACACC<br>GTCTACCTCCAGATGAACTCGCTCAAGCCCGAGGACACCG<br>CCGTCTACTACTGCAACGTCAACGTCGGCTTCGAGTACTG<br>GGGTCAGGGCACCCAGGTCACCGTCTCGTCGA	GeneArt (Thermo Fisher)
anti-botulinum toxin A nanobody (αBoNTANB) [2] [1]	ATGGCGGCC**CATCACCACCATCACCACCATCACCACCAT**<br>CATGGATCGGGTGGTGGTCTGGTCCAGGTCGGTGGCTCGC<br>TGCGTCTCTCGTGCGTCGTCTCGGGCTCGGACATCTCGGGC<br>ATCGCGATGGGCTGGTACCGTCAGGCTCCCGGCAAGCGTC<br>GCGAGATGGTCGCCGACATCTTCTCGGGTGGCTCGACCGA<br>CTACGCCGGCTCGGTCAAGGGTCGCTTCACCATCTCGCGC<br>GACAACGCCAAGAAGACCTCGTACCTCCAGATGAACAAC<br>GTCAAGCCCGAGGACACCGGTGTCTACTACTGCCGTCTCT<br>ACGGCTCGGGCGACTACTGGGGTCAGGGCACCCAGGTCA<br>CCGTCTCGTCG	Integrated DNA Technologies, Inc.

[1] The synthesized sequence includes a 10× Histidin tag (His tag; bold font) which was also codon-optimized for use in *U. maydis*. The actual start of the anti-botulinum toxin A nanobody (αBoNTANB) sequence is directly behind the His tag.

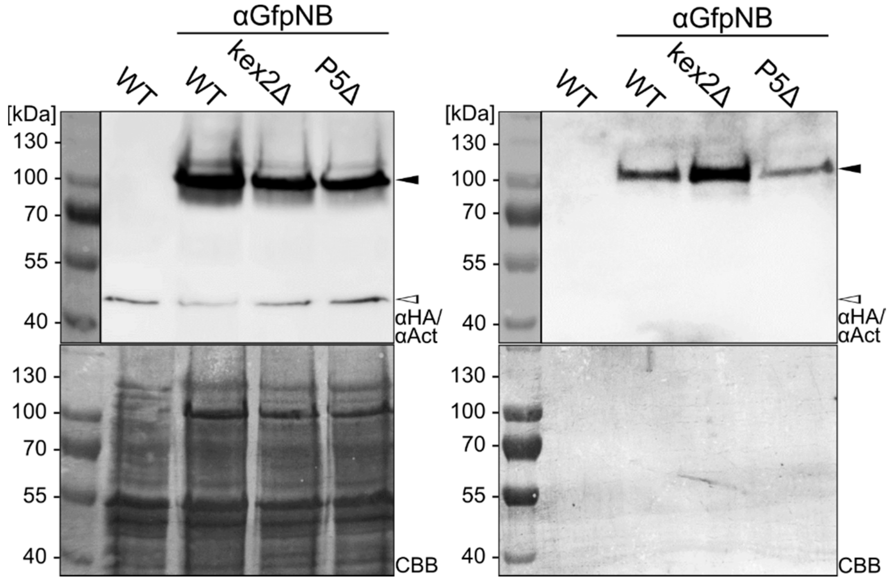

Figure S1. Expression and unconventional secretion of an anti-Gfp nanobody (αGfpNB) in *U. maydis*. The figure is a replicate of Figure panels 1**B** and 1**C**. The protein ladder has additionally been

included to clearly visualize the band sizes and a larger part of the Coomassie Brilliant Blue (CBB) stained membranes is provided.

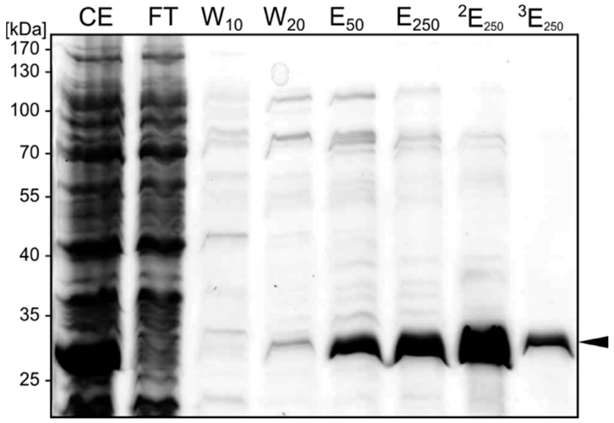

Figure S2. Expression and immobilized metal ion affinity chromatography (IMAC) purification of His-tagged Gfp (Gfp^H) produced in *Escherichia coli*. Identical volumes of the fractions were subjected to SDS-PAGE with subsequent CBB staining. CE, cell extract; FT, flow through; W, wash steps; E, elution fractions. Subscripts indicate concentrations of imidazole. Gfp^H is indicated with an arrowhead.

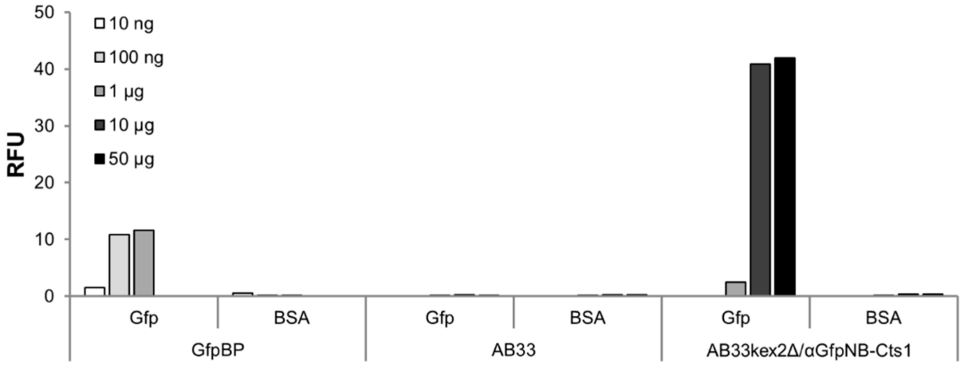

Figure S3. Positive control for nanobody enzyme-linked immunosorbent assay (ELISA) using commercial Gfp-binding protein. Detection of purified Gfp-strep-tag control protein (Gfp^S; IBA) in ELISA assays using indicated amounts of total cell extracts of strains AB33 (no nanobody) and AB33kex2Δ/αGfpNB-Cts1. As a positive control, detection was also performed using indicated amounts of commercial Gfp-binding protein (GfpBP, ChromoTek). Saturated signals were detected with 100 ng purified GfpBP and 10 µg *U. maydis* cell extracts containing αGfpNB-Cts1. Note that due to the presence of a C-terminal his-tag in GfpBP, the experimental setup has been changed and detection was performed with an antibody directed against the His tag (αHis).

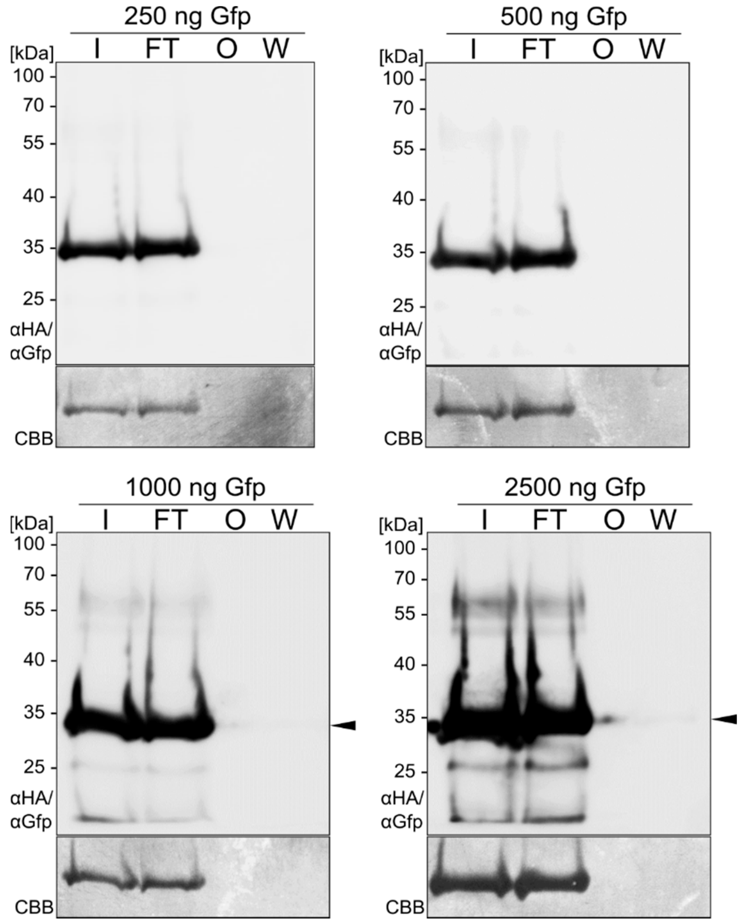

Figure S4. Titration of Gfp^{H} to determine suitable loading for Gfp-trap experiments. Indicated amounts of purified Gfp^{H} were incubated with chitin resin. The experimental setup was identical to the actual nanotrap experiments (Figure 5) just lacking cell extracts. The figure shows Western blots using primary αGfp and αHA antibodies. The membrane was stained with CBB after detection. Increasing the Gfp^{H} amount to 1.0 μg leads to inefficient washing and hence, unspecific bands in the elution fraction (indicated by arrowheads). Hence, no more than 500 ng input protein should be used in Gfp-trap experiments. I, input; FT, flow through; O, elution; W, last wash step.

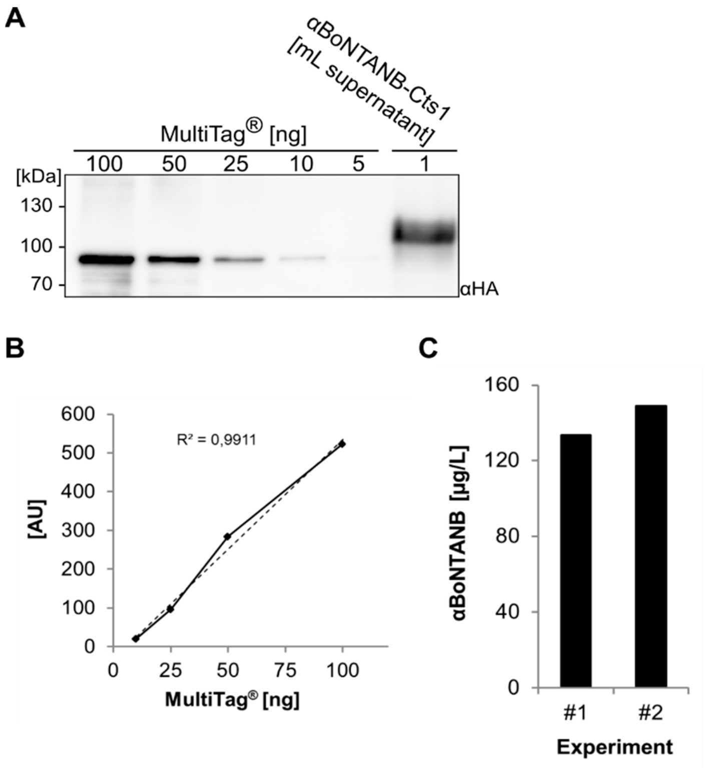

Figure S5. Quantification of αBoNTANB-Cts1 in *U. maydis* supernatants. (**A**) Representative Western blot of defined amounts of MultiTag® standard protein and trichloroacetic acid (TCA) precipitated supernatants of *U. maydis* AB33kex2Δ/αBoNTANB-Cts1 harvested 9 h post inoculation; (**B**) Standard curve from densitometric analysis of the MultiTag® standard protein depicted in **A**; (**C**) Amounts of αBoNTANB-Cts1 in µg/L calculated from Western blot results of 1 mL precipitated AB33kex2Δ/αBoNTANB-Cts1 supernatant. The experiment has been performed as a biological duplicate.

References (Supplementary Material)

1. Rothbauer, U.; Zolghadr, K.; Tillib, S.; Nowak, D.; Schermelleh, L.; Gahl, A.; Backmann, N.; Conrath, K.; Muyldermans, S.; Cardoso, M.C.; et al. Targeting and tracing antigens in live cells with fluorescent nanobodies. *Nature Methods* **2006**, *3*, 887–889.
2. Mukherjee, J.; Tremblay, J.M.; Leysath, C.E.; Ofori, K.; Baldwin, K.; Feng, X.; Bedenice, D.; Webb, R.P.; Wright, P.M.; Smith, L.A.; et al. A novel strategy for development of recombinant antitoxin therapeutics tested in a mouse botulism model. *PLoS ONE* **2012**, *7*, e29941.
